# Prehistoric Perspectives on “Others” and “Strangers”

**DOI:** 10.3389/fpsyg.2019.03063

**Published:** 2020-01-21

**Authors:** Anna Belfer-Cohen, Erella Hovers

**Affiliations:** ^1^The Institute of Archaeology, The Hebrew University of Jerusalem, Jerusalem, Israel; ^2^Institute of Human Origins, Arizona State University, Tempe, AZ, United States

**Keywords:** social behavior, paleolithic archaeology, socio-cognitive construct, social stereotypes, inter-group relationships, material culture proxies

## Abstract

Social “connectivity” through time is currently considered as one of the major drivers of cultural transmission and cultural evolution. Within this framework, the interactions within and between groups are impacted by individuals’ distinction of social relationships. In this paper, we focus on changes in a major aspect of social perceptions, “other” and “stranger.” As inferred from the archaeological record, this perception among human groups gained importance during the course of the Pleistocene. These changes would have occurred due to the plasticity of cognitive mechanisms, in response to the demands on behavior along the trajectory of human social evolution. The concepts of “other” and “stranger” have received little attention in the archaeological discourse, yet they are fundamental in the perception of social standing. The property of being an “other” is defined by one’s perception and is inherent to one’s view of the world around oneself; when shared by a group it becomes a social cognitive construct. Allocating an individual the status of a “stranger” is a socially-defined state that is potentially transient. We hypothesize that, while possibly entrenched in deep evolutionary origins, the latter is a relatively late addition to socio-cognitive categorization, associated with increased sedentism, larger groups and reduced territorial extent as part of the process of Neolithization. We posit that “others” and “strangers” can be approached from contextual archaeological data, with inferences as regards the evolution of cognitive social categories. Our analysis focused on raw material studies, observations on style, and evidence for craft specialization. We find that contrary to the null hypothesis the archaeological record implies earlier emergence of complex socio-cognitive categorization. The cognitive, cultural and social processes involved in the maintenance and distinction between “others” and “strangers” can be defined as “self-domestication” that is still an on-going process.

“*No man is an island entire of itself; every man is a piece of the continent, a part of the main;*” MEDITATION XVII, Devotions upon Emergent Occasions (*John Donne*)

## Introduction

While much research focused on the question of the emergence of “modern” cognition – a vague concept that is variably understood and therefore variably recognized (see, e.g., Belfer-Cohen and Hovers, 2010) – there is a growing realization that rudimentary forms of human cognition can be traced into deep prehistoric times (e.g., Deacon, 1989; Laland, 2017 and references therein). Research into human biological and social evolution has attempted to identify the role played by various cognitive aspects (e.g., Donald, 1993; Richerson and Boyd, 2000; van Schaik and Pradhan, 2003; Dunbar and Shultz, 2007; Andersson, 2011; Wynn and Coolidge, 2011, 2016; Grove, 2012; Jaffers, 2014; Dennett, 2016; Lombard and Gardenfors, 2017). Archaeologists of early prehistory directed their attention to the production and use of stone tools, focusing on planning depth, dexterity, and forms of teaching and learning. At the same time, primatologists, prehistorians, and paleoanthropologists developed lines of inquiry that focused on the capacity of the hominin brain to create, maintain and augment social relationships (e.g., Delagnes and Roche, 2005; Stout et al., 2008, 2011, 2019; Nowell and Davidson, 2010; Vaesen, 2012; Dunbar et al., 2014; Tomasello, 2014; Tomasello and Call, 2014; Dunbar and Shultz, 2017; Gärdenfors and Högberg, 2017; Herzlinger et al., 2017).

Plasticity of cognitive mechanisms is implicated as a major factor responsible for behavioral and cultural trajectories, as it both influences *and* is influenced by the way those have evolved over the course of prehistoric time. In fact, it seems unlikely that culture could have evolved without affecting cognition. Colagè and d’Errico (2018):4) argued that “…the brain [modifies] physiological, functional, and/or structural features as a consequence of experience and practice without any concomitant change at the genetic level” (see also discussion in Lotem et al., 2017).

In this context, the emergence of human sociality (Barrett et al., 2012) draws our attention to “social cognition” (SC) which emerges as an important element that influenced the structure, economy and culture of Paleolithic groups (e.g., Davies, 2016). SC constitutes people’s subjective interpretations of social situations as well as the concepts and cognitive processes whereby they were shaped (Korman et al., 2015; and see Thompson et al., 2016). Because of the assumed link between culture and SC, we also speculate that changes in cognitive social concepts, and thus in social relations, can be inferred from the cultural record of the Pleistocene.

The general question as to how prehistoric hominins structured their social relations is not a new one. Two fundamental, widely-discussed concepts in paleoanthropological research are those of “group” and “population” (e.g., Birdsell, 1958; Zhou et al., 2005; Gamble et al., 2014; White, 2017; Casari and Tagliapietra, 2018; and references therein). Attention was devoted to the variable forms of the organization of individuals into groups as the mechanisms that enabled rich and complex social configurations in extant and past hunter-gatherers (e.g., Grove, 2010; Hill et al., 2011; Grove et al., 2012; Bird et al., 2019; Domínguez-Rodrigo et al., 2019; Herzlinger and Goren-Inbar, 2019; Malinsky-Buller and Hovers, 2019).

In particular, shifts in group size and intra- and inter-group connectivity are currently considered as significant drivers of cultural transmission and cultural change (e.g., Premo and Scholnick, 2011; Derex et al., 2014, 2018; Stout, 2018; Greenbaum et al., 2019). The interactions within- and between- groups are complex processes that are impacted by the social roles of the individuals that comprise those units (Jenkins et al., 2018; Cacault and Grieder, 2019). These encompass the self-recognized place of an individual within her group, which in turn shapes “… the processes of formation of groups and changing membership of groups.” (Festinger, 1954: 135).

Here we discuss the SC concepts of “other” and “stranger,” fundamental in defining social interfaces within and between groups of hunter-gatherers, as they have shaped, to considerable degree, economic, mobility and territorial behaviors that can be inferred from the archaeological record. Yet, these concepts have rarely been addressed in archaeological discussion.

In the modern (and, mainly, post-modern…) discourse, “other” and “stranger,” are often used alternately. However, they are not synonymous. Dictionary definitions run:

“Other” – refers to a person or thing that is different or distinct from one already mentioned or known about^1^.

“Stranger” – a person who does not know a particular place or community or is not known in a particular place or community^2^.

Addressed from contextual archaeological data, our exploration is preliminary and inherently speculative, with inferences as regards the evolution of human sociality. As a caveat to the discussion we must add that the nature of prehistoric evidence clearly impedes archaeologists from discussing the socio-cultural process defined by cultural anthropologists and sociologists as “othering” or the concept of “The Other”.

The human social cognition system reflects the history of the genus *Homo*, potentially as far back as *Homo erectus* (∼1.8 million years ago), during which SC played a major part in constructing the human unique “niche” of adaptation to the external world (Laland et al., 2014; Davies, 2016). It seems to us that from the very beginning humans, “a highly intelligent creature who is tuned to the world’s complexity” (Davies, 2016:104), interacted with friends, “others,” “strangers,” and even family, through cultural “tool kits.” The latter would include shared social norms, predispositions and prejudices, being aware that “we are differently located in a shared world.” (*ibid*.). The active role of culture in shaping the social landscape of humans, in conjunction with genetic dictates and sometimes over-riding them, constitutes what one might define as “self-domestication.”

The property of “otherness” is inherent to one’s view of the surrounding world and to a large degree defines oneself (see definition above). In evolutionary terms the notion of being an “other” is an innate trait of human sociality, and is immutable. At a group-level, when a number of individuals consider themselves as “one,” vis-à-vis individuals or groups that do not belong to the “one,” “others” becomes a social cognitive construct. Still, there are degrees of social “otherness” that can be perceived within and between groups. Hunter-gatherer kinship terminologies (Chapais, 2014) make it psychologically possible to embrace non-kin members of one’s residential or task group, with whom one shares common history and beliefs, in order to accept them as kin. For example, Bird et al. (2019: 102), describing the Martu in the Western desert of Australia, state: “Many of the compound families in the 2005 census were actually multigenerational extended “classificatory kin” (e.g., older and younger “sisters” who share the same fictive kinship section, and are thus parallel “cousins”, but may have no close biological relationship).”

The concept of “stranger“is a trait of a complex society, an *emergent* social construct that is transient, since it depends on changeable cultural perceptions. In the words of Korman et al. (2015: 31), “This constant and reflexive updating of mental states presents a significant computational challenge, and people’s ability to conduct such rich and dynamic social interactions is one of the greatest achievements of human cognition.”

## A Suggested Diachronic Scenario

Some non-human primates appear to present behaviors that suggest that they may differentiate between “others” and “strangers.” To wit, the viability of a chimpanzee genetic pool (which is ≤300 individuals) is maintained by the relocation of fertile females among groups. When chimpanzees from those groups (“kin” or “others” in our terminology) encounter each other, the outcomes vary from skirmishes to friendship (see Tokuyama et al., 2019 for a recent discussion). This contrasts with encounters of groups from a different breeding pool (“strangers”), who fight until annihilation of one of the groups, including productive females.

Even when leaving aside the question of how deep the evolutionary roots of ape/chimpanzee aggression behavior might be (e.g., Ferguson, 2011; Sussman, 2013), there are a number of compelling arguments to support the view that the survival of human groups depended on the existence of social groupings of non-kin, even beyond “classificatory kinship” relationship. Viable mating networks, constituting some hundreds of individuals, are perhaps the most obvious such social units, within which interactions among “others” took place. Additional incentives for interacting with non-kin “others” may have involved the need to outcompete carnivores and gain safety through numbers. In many ecological contexts, low carrying capacity would preclude protracted and/or large aggregations, such that early hominins spent most of their time in small residential groups within an overall larger, even if mostly virtual, social structures (e.g., “extended groups,” Gamble, 1999). The latter constituted the pool for the “daily” grouping, enabling a flexible fission-fusion social structure, which was in turn constrained by ecology, technology and demography (Grove et al., 2012). Cautiously drawing inferences from extant populations, it is reasonable to assume that aggregations, namely large meetings that provided networking opportunities, were not random, as they had to be part of a basic behavioral routine, for example seasonal/annual aggregations, obligate exchange, etc. (e.g., Hovers, 1990; Bar-Yosef, 1997; Pearce and Moutsiou, 2014).

The frequencies of large group gatherings were dictated by population densities, such that lower densities would require more frequent aggregations. These meetings would have been of a limited duration. The reasons could be shortage of resources to sustain relatively large numbers of people. Since the meetings were scheduled, of a short duration and in an agreed-upon territory, there would be fewer opportunities for encroachment on the resources of the respective groups taking part in the aggregation. In ethnographic contexts, the fissioning of the aggregation stems from rising social tensions rather than environmental stress. When asked how people knew when to break up an aggregation, a !Kung informant replied that it happened when the women had no further information to share and started quarreling at the communal water hole (Konner, 2002).

Under these circumstances members of a particular group were likely aware of “us” or “we”, i.e., their own group members, as opposed to “others” with whom they had relatively brief social interactions. Under Paleolithic conditions (of sparse populations, spread over extensive territories) there would be only “us” and “others,” because every person, with whom one had any kind of interaction, belonged by default to the same reproductive network, even if not of the same basic social unit (extended family, kin and classificatory kin, residential unit). At all times, these social interactions involved kin and “others” but not “strangers.”

Because of the strong influence of culture on SC, changes in economic, demographic, spatial (and other) aspects of human culture introduced changes in perceptions of social relationship, i.e., in SC. Through time, one can observe a rise in residential group size and a reduction in territory sizes – both linked to improved means of production and associated demographic changes (Hayden, 1981; Gilman, 1984; Hamilton et al., 2007a, b). A larger group could serve as a viable genetic pool nearly on its own, without the need to meet members of other groups on a regular basis. Moreover, evolving complex societies would become more tethered to their geographic locations and more protective of their bounded resources and, in later periods, their surpluses. Encounters with individuals that did not belong to the same group would be less crucial for survival, and therefore would not be pre-scheduled or repetitive. On the other hand, when such encounters occurred, potential causes for inter-personal tensions would stem from economic interests, related to the availability, ownership and sharing of resources. Such encounters occurred between “strangers,” i.e., individuals or groups that did not share common history, cultural traditions, or behavioral patterns.

With the increase of global and local population sizes and change of economic mode, with the introduction of farming/agriculture, boundaries became less defined by clear-cut geographic features. Instead, “crowding” (e.g., by sedentism ensuing increased population densities) led to the emergence of socially constructed barriers. The definition of “strangers” lost its dependency on rare, random encounters. People came to consider as strangers also individuals they could meet on a daily basis but belonged on the other side of the social barrier.

The perception of “strangers” became even more nuanced through time. As networks of exchange (of both commodities and information) expanded, the rate of encounters with strangers increased, being beneficial to both sides. Under this premise some strangers, whose expertise and fields of knowledge complemented the ones present within a group, would be favored and would be more often tolerated by, if not accepted into, a given community. This differentiation became most obvious with the introduction of craft specialization and market economy. Hence it is in these later periods that we expect to find indications for “strangers” in the archaeological record.

The scenario presented above relies on theoretical, sociological and ethnographic knowledge, all engaged by researchers in attempts to structure a narrative of the evolution of human society and sociability. We find similarities with the question of children’s visibility in the archaeological record, a topic that, once raised, has burgeoned into a prolific field of inquiry (see, for example, Hammond and Hammond, 1981; Shea, 2006; Baxter, 2008; Chappell et al., 2013). The challenge herewith would be to evaluate the feasibility of archaeology to provide evidence based on the material culture record for the suggested scenario of the diachronic transformation of SC as regards “others” and “strangers.” On the evolutionary scale of prehistory, our questions should be formulated at the group rather than individual level.

## The Archaeological Evidence

An obvious question is which archaeological proxies can be used in addressing such questions. Given the scenario above, we suggest investigating aspects of the archaeological record that are often understood as markers of personal and group boundaries and alliances. Prominent among these are elements that denote territoriality, social identities, and within- and between-group relationship. Here we address examples from pertinent behaviors identified archaeologically.

The types of **raw materials** and their distributions within and between regions speak to the modes of procurement and transport and help to understand spatial dimensions of group interactions. At least in some instances, it is possible to discern between direct procurement (i.e., when a person/group traveled to the source and brought material back to a site) and forms of exchange. Each of these behaviors potentially bears implications for the type of interactions between groups in a given geographic space, namely, contacts with “others” or with “strangers.”

**Stylistic variations** within various categories of material objects have been argued to represent social identities at personal, group and regional levels. In some instances, stylistic variability can be tied to the fission-fusion social structure dynamic, specifically to the phase of aggregation when many “others” come together. It is sometimes possible to identify stylistic particularities that were used consciously as emblems, specifying group or population identities. By default, the absence of a shared emblem would denote one’s state of “stranger.”

The phenomenon of **craft specialization** pertains to differences in both style and production technology. Whereas experts perform with high levels of both conceptual and practical (*savoire-faire*) knowledge, average or novice practitioners possess the theoretical knowledge that is embedded in their material culture traditions, but would implement it poorly. Hence these two (broad) categories of skill are identified archaeologically (e.g., Karlin et al., 1993).

In a number of published case studies, the signature of local experts, who adhere to the raw materials, technological practices and styles of their group, has been recognized. Conversely (in particular during later prehistory) there are instances when the material record suggests that an artisan was not local, as expressed by the use of technology and style that had emerged and developed elsewhere. This would suggest a different type of social standing within the group, that of a “stranger.”

As our archaeological experience lies mainly within the Levantine record, we discuss some of the implications of the above scenario by looking closely at the details of selected case studies from the Levant. Our insights from the regional record are then contextualized into a broader geographic scope in the Discussion.

### Raw Material

**FIGURE 1:**
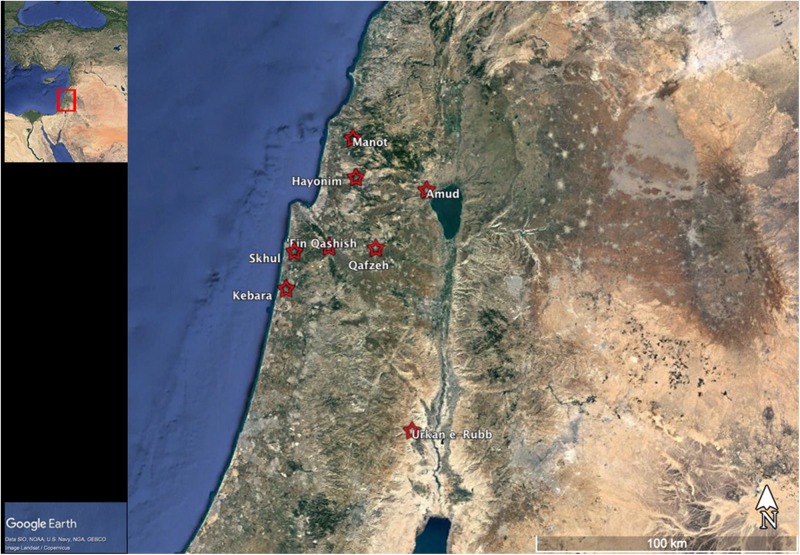
Map showing the location of sites mentioned in the text. Base map from Google Earth.

In the Levant (Figure 1), flint is the nearly exclusive raw material used for making stone tools (Goring-Morris et al., 2009). Its ubiquity on the landscape suggests that throughout time the optimal behavior of Levantine hominins was that of local procurement from nearby sources of suitable flint, embedded in their subsistence system. This pattern characterizes many Eurasian Paleolithic groups (e.g., Geneste, 1985; Féblot-Augustins, 1993, 2009). It has even been suggested that the same raw material sources/quarries had been recognized and utilized over tens of thousands of years (Gopher and Barkai, 2014; Finkel et al., 2019). If that scenario holds, it would suggest that (for each period) such locations may have served as places of meeting between “others,” i.e., individuals that belonged in a single mating system.

However, studies of raw material-related behaviors among Middle Paleolithic (MP; ca. 250,000–50,000 years ago) groups in the Levant suggest that hominins (both Neanderthals and modern humans) did not always opt for a “least effort” solutions for raw material requirements. At times, they obtained a variable (across sites) fraction of the raw material from additional, diverse sources, some of which were located at relatively long distances from the habitation sites. At Amud Cave (Figure 1; ca. 68,000–55,000 years ago), for example, this entailed a relatively significant, though non-systematic, transport from numerous, different sources located at distances of over 60 km. Notably, the techno-typological traits and the way of using the “exotic” raw material do not differ from those of the local flint, suggesting that the same people used the local and non-local sources (Ekshtain et al., 2017). Unexpectedly, the existence of a “buffer zone” in the central Galilee, located midrange between the local and the most distant outcrops, was revealed by this analysis. This zone remained unexploited by the Amud hominins, even though it contained many flint outcrops of reasonable quality (Ekshtain, 2015). The appearance and distribution of non-local raw material behavior in the Amud Cave assemblages can be interpreted as the outcome of direct procurement from distant sources. This would potentially entail encounters with groups of “others” within the same mating system. It would constitute an example of permeable social boundaries and the inclusion of the “other” within the economic and cognitive realm of the group. However, the presence of a “buffer zone” possibly demarcates a rigid boundary between groups that perceived each other as “strangers.”

The notion that the construct of “strangers” was activated in the social cognition of MP hominins gains support from another case study at the open-air site of ‘Ein Qashish (Figure 1; ca.70,000–60,000 years ago). Lithic raw materials were imported into the site from both local and more distant sources. Interestingly, the site’s occupants seem to have avoided exploiting available, good-quality sources located east of the site (Ekshtain et al., 2014). This pattern could not be explained through strictly economic aspects of raw material organization. Thus, Ekshtain et al. (2014) suggested that the decision of ‘Ein Qashish hominins to avoid exploitation of these sources speaks to social/cultural constraints imposed by territorial boundaries (e.g., Wilson, 2007) between groups identifying themselves as “strangers” to each other.

The different implications for social cognitive distinctions, associated with direct transport vs. indirect procurement through exchange, may be best considered when dealing with raw materials from distinct geographic sources. In the Levantine record, this can be done by looking at marine shells. Such items are known from MIS 5 (ca. 120,000–90,000 years ago) in Qafzeh and Skhul caves (Figure 1; Vanhaeren et al., 2006; Bar-Yosef Mayer et al., 2009) as well as in the later open-air site of ‘Ein Qashish (70,000–60,000 years ago) (Ekshtain et al., 2019). The relative proximity of the sites to the Mediterranean shorelines of their respective times of occupation and the sporadic appearance of shells in the relevant assemblages likely reflect direct transport rather than exchange (Bar-Yosef Mayer et al., 2009; Hovers, 2009), seemingly within the territory of a mating network (i.e., “others”).

A later example from the Epi-Paleolithic (24,000–11,500 years ago) site of Urkan e-Rubb IIa (Figure 1; ca. 17,000 years ago; Hovers and Marder, 1991) requires a more nuanced consideration. Located in the Jordan Valley, this site was a place of bead production, to which shells of selected genera and species were brought unmodified, cut and made into beads *in situ*. Because of the distance from the contemporaneous shoreline of the time (some 70 km to the west), and because of the large number of shells found in Urkan, this situation could be construed as indicative of exchange between groups, reflecting contacts with “strangers” beyond the group’s habitual territory. Two lines of evidence suggest differently: first, among the shells that reached the site very few belong to species that were not part of the repertoire of Epi-Paleolithic groups (Hovers et al., 1988; Bar-Yosef Mayer, 2005). These shells seem to have been collected due to mistaken identification. If the Urkan shells were retrieved by exchange, it is likely that the mistake would be discovered during negotiations. Thus, these specimens reflect collectors’ error, indicating that the site’s occupants themselves traveled to the shores of the Mediterranean. Moreover, the lithic assemblage of Urkan bears striking similarities to those from sites located in a specific part of the east Mediterranean coast (Hovers et al., 1988; Hovers and Marder, 1991). In the present context of discussion, the archaeology seems to imply that the occupants traveled to the shoreline within their own territory, and that they encountered “others,” rather than “strangers.”

### Style

Stylistic characteristics are widely accepted as expressions of individual or group identity (Sackett, 1990; Hegmon, 1992; McElreath et al., 2003; McGuire and Hildebrandt, 2018; see papers in Wobst, 1977; Stark, 1998). To bear on the cognitive differentiation between “other” and “stranger” at the population level, we turn to a striking example of its expression in the Levantine record.

The “Levantine Aurignacian” is a short-lived (37,000–34,000 years ago) cultural entity that existed only in the northern part of modern Israel and along the Lebanese coast. It is a unique phenomenon in the Upper Paleolithic (UP; 48,000–24,000 years ago) sequence of the Levant in that it shows greater similarity to the West European “classic Aurignacian” cultural entity (dated there to ca. 40,000–27,000 years ago) than to the local archaeological entities that immediately precede or follow it (Belfer-Cohen and Goring-Morris, 2014). This similarity is seen in both its chipped stone typo-technology and its non-lithic objects. Among the latter are personal ornaments such as tooth pendants as well as bone and antler worked items (Goring-Morris and Belfer-Cohen, 2006). Both the European Aurignacian and the Levantine Aurignacian shared complex technical concepts of antler working as opposed to simpler bone-working technologies.

A unique feature of the Levantine Aurignacian is the occurrence of notched bones (n = 15; typically, gazelle scapulae as well as a single hyoid bone) from the Aurignacian levels of Hayonim, Manot, Kebara (and possibly Emireh) caves (Figure 1), all located in northern Israel. Contrary to European Aurignacian sites, where similar items were reported as sporadic finds per site/assemblage, the assemblage from Hayonim Cave is relatively large (n = 9; Tejero et al., 2018). A careful technological analysis demonstrated that the notches constitute intentional markings. Whereas other modified bone objects in the same assemblages, mainly awls and “chisels” intended probably for mundane use, were minimally modified, the notched scapulae were shaped through a complex and specific technique, rarely observed in the Aurignacian techno-complex in Europe.

The difference between the Levant and Europe *vis-*a-*vis* notched items is expressed in the selected animal taxa, the raw materials and anatomical elements used, and in the types of “decorated” objects. In the European Aurignacian notches were made on bone, antler and ivory pieces deriving from reindeer, red deer, bovid, and mammoth; the marks occur on antler, bone splinters, personal ornaments and on “utilitarian” tools such as polishers. In contrast, notched pieces in the Levantine Aurignacian are highly uniform and homogenous, with gazelles being the only animal species selected and with an almost exclusive use of a single anatomical element, the scapula.

The standardization of their production procedures as well as their relative concentration in Levantine Aurignacian assemblages indicate that these items were unique features of the regional record. It was suggested that they served as an emblem of the Levantine Aurignacian (Tejero et al., 2018). Such items possibly reflect the strong ties between various Levantine Aurignacian communities by serving as a marker of “us” and “others” (i.e., biological and “classificatory” kin), differentiating them from the surrounding population of “strangers” (e.g., the native Ahmarian cultural entity)^3^, who did not share the social and cultural worldviews expressed through this particular cultural item.

Tooth pendants, recovered from the very same sites of the short-lived Levantine Aurignacian, illuminate another facet of the social cognitive distinctions between “others” and “strangers.” By way of example, we use the assemblage from the same layers at Hayonim Cave (Belfer-Cohen and Bar-Yosef, 1981). In contrast to the notched pieces, tooth pendants were never made on gazelle teeth, but on the vestigial canines of (mostly) male red deer (*Cervus elaphus*), and were shaped by a particular technique. Both the manufacture marks and the use-wear signs point toward personal ornaments. Some of the modification processes differed from those observed in other Levantine Aurignacian sites (Tejero et al., submitted).

In the European Aurignacian record, the exploitation of mammal teeth – including in some cases human teeth – became a common practice, probably playing a role in the symbolic sphere of these hunter-gatherer groups. Different teeth (incisors, canines, premolars and molars) of a large spectrum of herbivores (e.g., reindeer, red deer, horse, bison, goat) and carnivore (e.g., bear, wolf, fox) species were selected for the manufacture of these ornaments. The flexibility seen in the techniques employed for ornament production is expressed by the emergence of region-specific (albeit interconnected) characteristics (Vanhaeren and d’Errico, 2006).

The use-wear of the Levantine and European Aurignacian pierced teeth implies identical utilization of the objects, suggesting shared symbolic practices. The *in situ* production of the pendants, demonstrated at least for some of the Hayonim items, reinforces this suggestion. This contrasts significantly with the archaeological manifestations of the locally-rooted Ahmarian techno-complex. Thus, accepting that the pendants were markers of the Aurignacians, their similarity in the Levant and in Europe is striking. It lends support to the notion of the Levantine Aurignacian as an incursion from Europe that is linked to its geographic origins through cultural tradition. The pendants therefore suggest that the Aurignacian populations were “others” within a Mediterranean meta-population, with a shared history that arose before the “migration” of Aurignacian groups into the Levant, and which defined them as “strangers” to the local Ahmarian groups of the eastern Mediterranean coast. Thus, the differentiation was not necessarily constrained or dictated by geographic distance. For the Levantine Aurignacian, “otherness” and “strangeness” seem to have been first and foremost constructs of social cognition.

### Craft Specialization

We hypothesized (see above) that encounters with strangers should become more apparent in the archaeological record of later periods. We also suggested that their presence can be detected through identification of the products of expert artisans bringing with them knowledge that is new to the local groups.

Although expert lithic knappers may have existed in the Levant as early as the Acheulian, it is of note that their activities are understood as those of local artisans acting within the context of a (sometimes large) residential group (Herzlinger and Goren-Inbar, 2019) of kin (in the extended sense of Bird et al., 2019). Comparable evidence is lacking for most of the Levantine Paleolithic record (possibly due to research/preservation constraints), yet the presence of expert knappers has been recognized in other Paleolithic records, such as the UP Magdalenian culture (ca. 15,000 years ago), in Etiolles, France (Pigeot, 1990 and references therein). There, too, expert knapping occurred within a residential context, and experts are regarded as models for novice knappers.

The context of activities of expert knappers in the Levant seems to have changed in the Pre-Pottery Neolithic B (ca. 10,500–8,400 years ago), possibly as part of the Neolithization processes. The “naviform” mode of flaking stone, designed for the production of long and thin blades, was a pan-Levantine phenomenon (Bar-Yosef and Belfer-Cohen, 1989). Naviform products (arrowheads and sickle blades) expertly produced on non-local raw material, were found together with items sharing the same regional conceptual knowledge yet crudely knapped on local raw material (Barzilai and Goring-Morris, 2007; Khalaily et al., 2007, 2013; Barzilai, 2010; Mitki, 2015). A common explanation of this phenomenon is that the well-made items were introduced into the local communities from outside the region. We maintain that those were produced by expert knappers rather than traded, because of the presence at the sites of production debris besides the finished products (*ibid*.). The material cultural record shows that naviform technology first emerged and evolved in the northern Levant (e.g., current-day central and eastern Turkey, northern Syria), and that its craftsmanship in the north was overall more refined than in the southern Levant. We therefore posit that, heralding from the northern Levant, the expert knappers of naviform items would be perceived as “strangers” by the local communities.

## Discussion

Chimpanzees and humans both appear to harbor concepts of “strangers,” which may be attributed to a shared evolutionary origin. The encounters of chimpanzee groups with “strangers” are rare, and were reported rather sparsely (e.g., Goodall, 2010; Wilson et al., 2014), because the spatial packing of chimpanzee groups on the landscape typically does not bring groups from different breeding networks into contact. Based on current evidence (which is incomplete for Pleistocene hominins due to the vagaries of time), in human society this SC construct has changed from that of the chimps (and presumably, an early ancestor) during the course of the Pleistocene. We identify the main changes in that the notion of “strangers” is enacted upon constantly in the context of large social networks, and in that it is fluid and transient.

Nearly all researchers agree that hominins, unlike perhaps other species, have found cognitive and social scaffoldings that enable them to operate within very large groups, cross-culturally. It is also clear that at some point in human cultural and social evolution, one’s recognition of “group size” shifted from census numbers to a social perception. Notwithstanding any biological limitations on group size, humans acquired a cognitive flexibility that enabled them to first, enlarge the biological and social perception of kin and, secondly, to categorize their social world as one of stable (“kin”/“classificatory kin”/“others”) and transient relationships (“strangers”). The latter could be remodeled contextually (i.e., politically, economically or culturally) according to shifting circumstances. Already Isaac (1972) wrote that once culture became more complex and comprehensive and social rules became more structured, it was more likely that internal isolating mechanisms would develop. Gilman (1984) specifically related to shifting circumstances when explaining the difference between the MP vs. UP social interactions. “Thus, as technique improved, relations between groups would become more problematical. [In the earlier periods] the give-and-take of mutual aid would have been so essential that it would have known no social boundaries…” (Gilman, 1984:122). In contrast, in the cultural world of the UP, improved technology led to higher group densities, such that more neighbors became available yet there were fewer occasions on which help from neighboring groups would be required to mitigate environmental risks. “The clear solution to this shift in the balance of a group’s interests would be to restrict the scope of its alliances.” (*ibid.*). Similar to Stiner and Kuhn (2006), we interpret the UP pattern to suggest higher degrees of connectivity between groups belonging to the same cultural environment, yet we propose that such connectivity was structured differentially across geographic space, with “strangers” located more distally to a given group than were “others.” We argue that these large UP groups could not have formed without the emergence of the SC construct of “strangers.”

Furthermore, the archaeological evidence allows us to identify earlier-than-expected trends in the emergence of the social cognitive constructs related to inter-personal/intra-group and inter-group behaviors. There is indirect evidence that large social groupings, with their implied categorization of social relationships, emerged earlier than the UP, thus our null hypothesis must be rejected.

We have focused our archaeological discussion on Levantine case studies, using information from lithic raw material, bone tools and personal ornaments. When contextualized against the broader archaeological records of the respective periods outside of the Levant, archaeological data provide insights of similar social cognitive constructs. For example, larger transport distances of raw material entailed, almost by default, awareness and recognition of “strangers.” Long transport distances of obsidian in eastern Africa (>200 km and sometimes >300 km; Blegen, 2017; Merrick et al., 1994; Negash and Shackley, 2006) are reported from the early Middle Stone Age (MSA; ca. 320,000–50/40,000 years ago) onward. These data could be interpreted to reflect large home ranges of “others” acting within a social/cultural group, interacting directly at the obsidian sources. More likely, this pattern should be attributed to indirect procurement (e.g., Merrick et al., 1994; Tryon and Faith, 2013) through the agency of “strangers.” Indeed, it has been argued that this very pattern reflects the increase in spatial extent of the social networks, as is the case with modern hunter-gatherers (Pearce, 2014; Pearce and Moutsiou, 2014). Similar behaviors may explain the distances of obsidian transport in the Caucasus (see discussion in Doronicheva and Shackley, 2014), which in some cases were >500 km during the late MP and the UP (Frahm et al., 2019). The late MP in Eurasia may be the first time when “strangers” become an element of the social structure, within networks of partial connectivity greatly contributing to the growth and evolution of human culture at large (Derex and Boyd, 2016; Premo, 2016; Derex et al., 2018). In fact, this may be the continuation of a trend that had started in the Middle Pleistocene (e.g., Rolland, 2004; Kuhn and Stiner, 2019) and gradually increased through time, as indicated by the Eurasian UP record in general (Stiner and Kuhn, 2006), and the Levant specifically (see above).

A known phenomenon in the south Levant, of sites dating to Late UP [Epipalaeolithic] and Neolithic times, is that of obsidian sourced to central and northern Anatolia. This is parsimoniously explained as evidence of down-the-line trade or exchange (see Ammerman, 1979 for a case study outside the Levant) involving “others” and/or “strangers,” which grew in scope with the process of Neolithization. Indeed, the evidence for the activities of non-local expert knappers in south Levantine Early Neolithic villages corroborates such interpretations. In fact, this may be the culmination of a trend that had started ca. 400,000 years ago, in the late Middle Pleistocene (e.g., Kuhn and Stiner, 2019) and gradually increased through time, as indicated by the Eurasian UP record in general (Stiner and Kuhn, 2006; and see above).

As we understand it, the trend is consistent with Isaac (1972) and Gilman (1984) insights that as economy and technology became more complex, it required constant evaluation of social cognitive rules and their ongoing restructuring within the respective cultural contexts. The separation between “us” and “others” *vis-a-vis* “strangers” would be instrumental in alleviating “scalar stress” (as defined by Johnson, 1982) within a large group. The breaking down of larger and growing social units into smaller, conceptually “manageable” ones, would require the creation of social stereotypes that one could allude to (see also Cohen, 1985). This is a process that has been observed in historical and extant societies and apparently is still ongoing. A historical example is the ancient Greek worldview, by which a social universe was divided almost by default into two: “us” (and all related “others”), meaning familiar, Greek-speaking individuals/political entities; and the rest of the world populations, “strangers,” i.e., unfamiliar individuals/political entities that did not speak the language, all of them stereotyped as “Barbarians.” In fact, to this day group identity creates cognitive social and economic biases and stereotypes that affect venues of modern life (e.g., Cacault and Grieder, 2019). In this sense, SC constructs that have emerged in deep prehistoric times affect many aspects of our modern lives, in an ongoing process of self-domestication.

## Author Contributions

Both authors perceived and wrote the manuscript.

## Conflict of Interest

The authors declare that the research was conducted in the absence of any commercial or financial relationships that could be construed as a potential conflict of interest.

## Footnotes

1 https://en.oxforddictionaries.com/definition/other

2 https://en.oxforddictionaries.com/definition/stranger

3 The Ahmarian differs from the Aurignacian mainly in its characteristic flint tools and the near absence of bone and antler artifacts. It is dated to 46,000–30,000 years and accordingly is contemporaneous with the Levantine Aurignacian (Alex et al., 2017; Goring-Morris and Belfer-Cohen, 2018).
